# Comparative characterization of volatile components in commercial Huangjiu with different sweetness classifications using electronic nose, HS-SPME-GC-MS and GC-IMS

**DOI:** 10.3389/fnut.2026.1941350

**Published:** 2026-10-02

**Authors:** Ze Zheng, Dongxue Qi, Xiaolei Tang, Jianan Qi, Yiwei Zhao, Yingqian Li, Zhen Dong, ZiYing Gao, Di Zou, Bin Qi, Li Liu

**Affiliations:** 1 School of Pharmacy, Changchun University of Chinese Medicine, Changchun, China; 2 Research Center of Traditional Chinese Medicine, The Affiliated Hospital to Changchun University of Chinese Medicine, Changchun, China; 3 Department of Statistics and Data Science, Southern University of Science and Technology, Shenzhen, China; 4 Quality Certification Department, Jilin Provincial Product Quality Supervision and Inspection Institute, Changchun, China; 5 Department of Nephrology, The Affiliated Hospital to Changchun University of Chinese Medicine, Changchun, China

**Keywords:** GC-IMS, Huangjiu, HS-SPME-GC-MS, Electronic nose, volatile flavor compounds

## Abstract

**Background:**

Huangjiu has diverse flavor characteristics across four official sweetness classifications, and complementary analytical measurements can provide a more detailed comparison of volatile profiles among commercial products.

**Methods:**

Commercial Huangjiu samples representing dry, semi-dry, semi-sweet, and sweet types from the same manufacturer were characterized using an electronic nose, HS-SPME-GC-MS, and GC-IMS.

**Results:**

E-nose responses indicated differences in response patterns among the four commercial products. HS-SPME-GC-MS tentatively identified 56 volatile compounds, while GC-IMS tentatively identified 54 volatile compounds, excluding the internal standard. The two platforms were complementary: GC-MS provided broader compound coverage, while GC-IMS showed enhanced sensitivity toward low-molecular-weight volatile compounds. Comparative analysis revealed differences in volatile composition and identified differential volatile compounds associated with sample separation in the fitted models.

**Conclusion:**

These findings provide a comparative chemical basis for understanding the quality characteristics of commercial Huangjiu with different sweetness classifications and demonstrate the value of integrating complementary analytical techniques for fermented food characterization and quality evaluation.

## 1 Introduction

Huangjiu, also known as Chinese rice wine, is a traditional fermented alcoholic beverage in China and is recognized as one of the world’s three oldest wines, alongside beer and grape wine. Huangjiu is widely consumed in China, serving not only as a culinary seasoning but also as an auxiliary material in traditional Chinese medicinal processing, with broad application prospects (1).

Huangjiu (yellow rice wine) comprises a wide variety of types. Based on total sugar content, Huangjiu can be classified into four categories: dry, semi-dry, semi-sweet, and sweet. Dry-type Huangjiu, also known as Yuanhong (YH), contains ≤ 15.0 g/L of total sugar; semi-dry Huangjiu, also referred to as Jiafan (JF), contains 15.1–40.0 g/L of total sugar; semi-sweet Huangjiu, also known as Shanjiang (SL), contains 40.1-100 g/L of total sugar; and sweet Huangjiu, also termed Xiangxue (XX), contains > 100.0 g/L of total sugar. The differences in sugar content among Huangjiu types are primarily attributed to the regulation of saccharification and the timing of fermentation termination during brewing, as well as variations in raw materials and microbial communities (2). Residual sugar is the key determinant of sweetness in Huangjiu. Flavor differences arise from the combined effects of raw material selection, production techniques, and climatic conditions during brewing, resulting in substantial diversity in the types and concentrations of volatile components in Huangjiu (3). Volatile components constitute a critical component of Huangjiu flavor, and their composition and concentrations are influenced by factors such as raw materials, microbial communities, fermentation temperature, and post-fermentation conditions. To date, the major volatile compounds identified in Huangjiu include alcohols, esters, organic acids, aldehydes, ketones, terpenes, and other minor constituents (2, 4, 5). These components play a crucial role in determining the unique flavor, quality, and style of Huangjiu. For instance, phenylethanol is an important aromatic compound that imparts a pleasant floral (rose-like) aroma to Huangjiu (6). Ethanol is the primary fermentation product, accompanied by higher alcohols such as isopentanol and phenylethanol. These alcohols contribute significantly to the overall flavor and aroma profile of Huangjiu.

Electronic nose (E-nose) technology and gas chromatography–mass spectrometry (GC–MS) have been applied to characterize the volatile profiles of alcoholic beverages (7, 8). Compared with traditional sensory evaluation methods, e-nose technology overcomes the limitations of human perception, such as ambiguity, subjectivity, and variability, while also reducing the complexity associated with conventional chromatographic analyses. It has been widely applied in the food industry (9, 10). GC–MS is commonly used for the separation, identification, and quantification of volatile organic compounds in wine (11). GC–MS is often combined with headspace solid-phase microextraction (HS-SPME), which offers advantages such as operational simplicity, high sensitivity, and ease of automation, enabling rapid and accurate analysis (12, 13). Gas Chromatography-Ion Mobility Spectrometry (GC-IMS) is an emerging analytical technique for trace gas detection and ion characterization, based on differences in gas-phase ion mobility under a constant electric field, and exhibits high sensitivity toward electronegative and proton-affinitive substances (14, 15). GC-IMS integrates the advantages of gas chromatography (GC) and ion mobility spectrometry (IMS), offering high resolution and ultra-high sensitivity, thereby enabling both quantitative and qualitative analysis of trace VOCs, particularly for isomer discrimination (16). Currently, GC-IMS has been widely applied in the detection of pesticide residues in fruits and vegetables, identification of food additives, flavor analysis of distilled spirits, assessment of honey maturity and origin, as well as characterization and freshness evaluation of meat and meat products (17–22).

Previous research has already examined aroma compounds across different Huangjiu types. Yu et al. analyzed four types of Chinese rice wine using GC–MS and GC–olfactometry, detecting 61 aroma compounds and identifying 18 as key aroma compounds (23). A subsequent study quantified 20 esters in four typical Huangjiu types and demonstrated sensory synergy between selected ester pairs, including ethyl lactate and ethyl acetate (24). These findings establish a basis for understanding compound composition and aroma interactions across Huangjiu types. Building on this work, the present study addresses a complementary, product-level question: how E-nose response patterns and the volatile profiles obtained by HS-SPME-GC–MS and GC-IMS jointly characterize four commercial products representing different sweetness classifications.

Previous studies have established the utility of E-nose, HS-SPME-GC–MS, and GC-IMS for characterizing fermented beverages. Building on these approaches, the present study examines how complementary analytical measurements characterize differences among four commercial Huangjiu products covering the official sweetness classifications. Specifically, E-nose responses were used to compare overall volatile-response patterns, while HS-SPME-GC–MS and GC-IMS provided complementary information on individual volatile constituents and their distributions among products. The contribution of this study lies in the integrated, product-level comparison and the resulting compositional information, rather than methodological innovation. The objective was to characterize the volatile profiles of the four selected products and assess the complementary information provided by the analytical platforms. This comparison was not designed to isolate the causal effect of sugar content or establish universal markers of sweetness categories.

## 2 Materials and methods

### 2.1 Samples and chemicals

Four commercially available Huangjiu products representing the four official sweetness classifications in the Chinese national standard (dry, semi-dry, semi-sweet, and sweet) were purchased from the same manufacturer (Shaoxing Jianhu Brewing Co., Ltd., Shaoxing, China) through the JD.com platform. The four products were selected to provide a comparison of commercially available Huangjiu covering the four sweetness classifications within one manufacturer. This sampling strategy limits between-manufacturer variability, although product-specific characteristics remain inseparable from sweetness classification. All samples originated from the same commercial source and were stored at 4 °C until analysis.

Octanol (analytical grade) was obtained from Aladdin; Sodium chloride (NaCl) was supplied by Tianjin Kemio Chemical Reagents Co., Ltd.; purified water (Wahaha) was used throughout the experiments. N-alkyl ketones including 2-butanone, 2-pentanone, 2-hexanone, 2-heptanone, 2-octanone, and 2-nonanone (all analytical grade), were purchased from Aladdin; 2-octanol (chromatographic grade) was obtained from Aladdin; nitrogen gas (99.999% purity) was used as the carrier gas.

Three independent production batches were obtained for each commercial Huangjiu product, and samples from different batches were not pooled before analysis. For HS-SPME-GC–MS and GC-IMS, three aliquots were independently prepared and analyzed per batch. The measurements within each batch were averaged, yielding three batch-level observations per product for these analyses. For E-nose analysis, five measurements were performed per batch. Measurements with the same replicate index were averaged across the three batches, yielding five averaged response profiles per product. These five profiles were not treated as independent biological replicates, and the E-nose results were interpreted descriptively and exploratorily.

### 2.2 Analysis of physicochemical parameters

Total sugar and alcohol contents were determined according to GB/T 13662–2018. Total sugar was measured using the Lane–Eynon method for XX and SL (Section 6.2.1) and the potassium ferrocyanide titration method for YH and JF (Section 6.2.2). Samples were hydrolyzed with hydrochloric acid at 68–70 °C for 15 min, cooled, neutralized, and diluted to volume before titration. Methylene blue was used as the endpoint indicator, and total sugar was expressed as glucose equivalents (g/L). Alcohol content was determined using an alcoholmeter after distillation and expressed as percentage alcohol by volume at 20 °C.

### 2.3 E-nose analysis

E-nose analysis was performed following the method described by Wu et al. (25) with minor modifications. Briefly, 5 mL of each Huangjiu sample was transferred into a 30 mL headspace vial, sealed, and equilibrated at 25 °C for approximately 2 h to ensure sufficient release of volatile components and headspace equilibrium. The sampling needle was directly inserted into the vial for headspace aspiration. The measurement conditions were as follows: sensor preheating/cleaning time, 20 min; inter-sample cleaning time, 60s; detection time, 100 s; injection flow rate, 10 mL/min; operating temperature, 25 °C. Five measurements were performed per production batch, and response profiles were averaged across batches as described in the Samples and Chemicals section. The electronic nose sensors and their representative sensitive compounds are listed in Table 1.

**Table 1:** Electronic nose sensors and corresponding representative sensitive compounds.

Sensor name	Main response substances
S1	Propane, smoke
S2	Carbon compounds
S3	Inflammable air
S4	Sulfide
S5	Nitrogen compounds
S6	Aldehydes
S7	Combustible gas
S8	Liquefied gas
S9	Alkanes, alcohols
S10	Hydrogen-containing gas
S11	Carbon monoxide alkanes
S12	Methane, liquefied gas
S13	Shorter chain-alkane
S14	Methane, gas, smoke
S15	Carbon substances, alcohols
S16	Hydrogen sulfide
S17	Ammonia, amines
S18	Toluene, acetone, ethanol

### 2.4 HS-SPME-GC-MS analysis

This method was adapted from Wang et al. (26) with modifications for Huangjiu sample analysis. Volatile compounds in Huangjiu were analyzed using a Shimadzu gas chromatography–mass spectrometry (GC-2010 coupled with QP-2010 plus; Shimadzu, Japan) equipped with an AOC autosampler. Semi-quantitative analysis was performed using 2-octanol as an internal standard. The relative concentrations of volatile compounds were estimated based on the peak area ratio between each analyte and the internal standard. Subsequently, 1.0 g of solid sodium chloride was added to the headspace vial to promote the salting-out of volatile compounds. The vial was sealed and placed in the autosampler. Each sample was analyzed in triplicate. Volatile compounds were extracted using a divinylbenzene/carboxene/polydimethylsiloxane extraction head (DVB/CAR/PDMS, 50/30 μm, Supelco, USA). The extraction conditions were as follows: incubation temperature, 80 °C; incubation time, 15 min; extraction time, 15 min; desorption temperature, 220 °C; desorption time: 1 min; splitless injection mode; fiber conditioning temperature: 250 °C; and conditioning time, 10 min. Separation was achieved using an HP-INNOWax capillary column (60 m × 0.25 mm × 0.25 μm, Agilent). The carrier gas flow rate was 1.0 mL/min, and the injector temperature was set at 220 °C. MS conditions were as follows: electron ionization energy, 70 eV; ion source temperature, 230 °C; and scan mode over a mass range of 50–500 m/z. Volatile compounds were tentatively identified by comparison with the NIST 11 mass spectral library (match factor >90%), followed by manual inspection of the corresponding spectra. Therefore, compound identification should be regarded as tentative unless otherwise stated.

### 2.5 GC-IMS analysis

The analysis was performed using a GC-IMS system (FlavourSpec, G.A.S, Dortmund, Germany) equipped with an MXT-WAX capillary column (30 m × 0.53 mm, 1.0 μm; Restek, Bellefonte, PA, USA). The column temperature was set at 60 °C. The GC-IMS system was equipped with an automatic headspace sampler (CTC, Analytics, AG, Zwingen, Switzerland). A 1 mL sample was transferred into a 20 mL headspace vial, followed by the addition of 10 μL of 100 ppm 2-octanol as an internal standard. After incubation at 60 °C for 20 min, the headspace sample was injected. Each sample was analyzed in triplicate. Following incubation at 60 °C for 20 min, 200 μL of headspace was injected using a syringe heated to 85 °C in splitless mode, with an agitation speed of 500 r/min. High-purity nitrogen (≥99.999%) was used as the carrier gas. The carrier gas flow rate was maintained at 2.0 mL/min for 2 min, increased linearly to 10.0 mL/min over 8 min, and further increased to 100.0 mL/min over 10 min, followed by a 20 min hold. The total chromatographic run time was 40 min. The injector temperature was set at 80 °C. The analytes were ionized in the IMS ionization chamber using a ^3^H source in positive ion mode. The generated ions were driven into a 53 mm drift tube under an electric field strength of 500 V/cm at 45 °C. High-purity nitrogen (≥99.999%) was used as the drift gas at a flow rate of 75.0 mL/min. Retention indices were calculated based on retention times, and qualitative analysis was performed by comparison with the GC retention index database (NIST 2020) and the IMS drift time database integrated into VOCal software (see Table 1).

### 2.6 Data analysis

VOCal software was used in conjunction with the Reporter, Gallery Plot, and Dynamic PCA plugins to generate three-dimensional spectra, two-dimensional spectra, differential spectra, fingerprint spectra, and PCA plots of volatile components for comparative analysis of volatile organic compounds among samples. For HS-SPME-GC–MS, compounds were tentatively identified using the NIST 11 mass spectral library, with a matching score >90%. For GC-IMS, tentative identification was based on retention indices and drift times using the NIST 2020 retention index database and the IMS drift time database integrated into VOCal software. Radar charts were generated using Origin 2020. Orthogonal partial least squares discriminant analysis (OPLS-DA), variable importance projection (VIP) analysis, permutation testing, and bivariate plots were performed using SIMCA 14.1. Data processing was performed using Excel 2010, whereas data analysis, principal component analysis (PCA), and heatmap generation were conducted using Origin 2020. The heatmap in Figure 1 was generated in Python using the mean semi-quantitative concentrations in Table 2. Values were standardized within each compound using *Z*-scores calculated with the sample standard deviation. Entries reported as a dash were assigned zero for this descriptive analysis. Product profiles were hierarchically clustered using Euclidean distance and average linkage.

**Figure 1:**
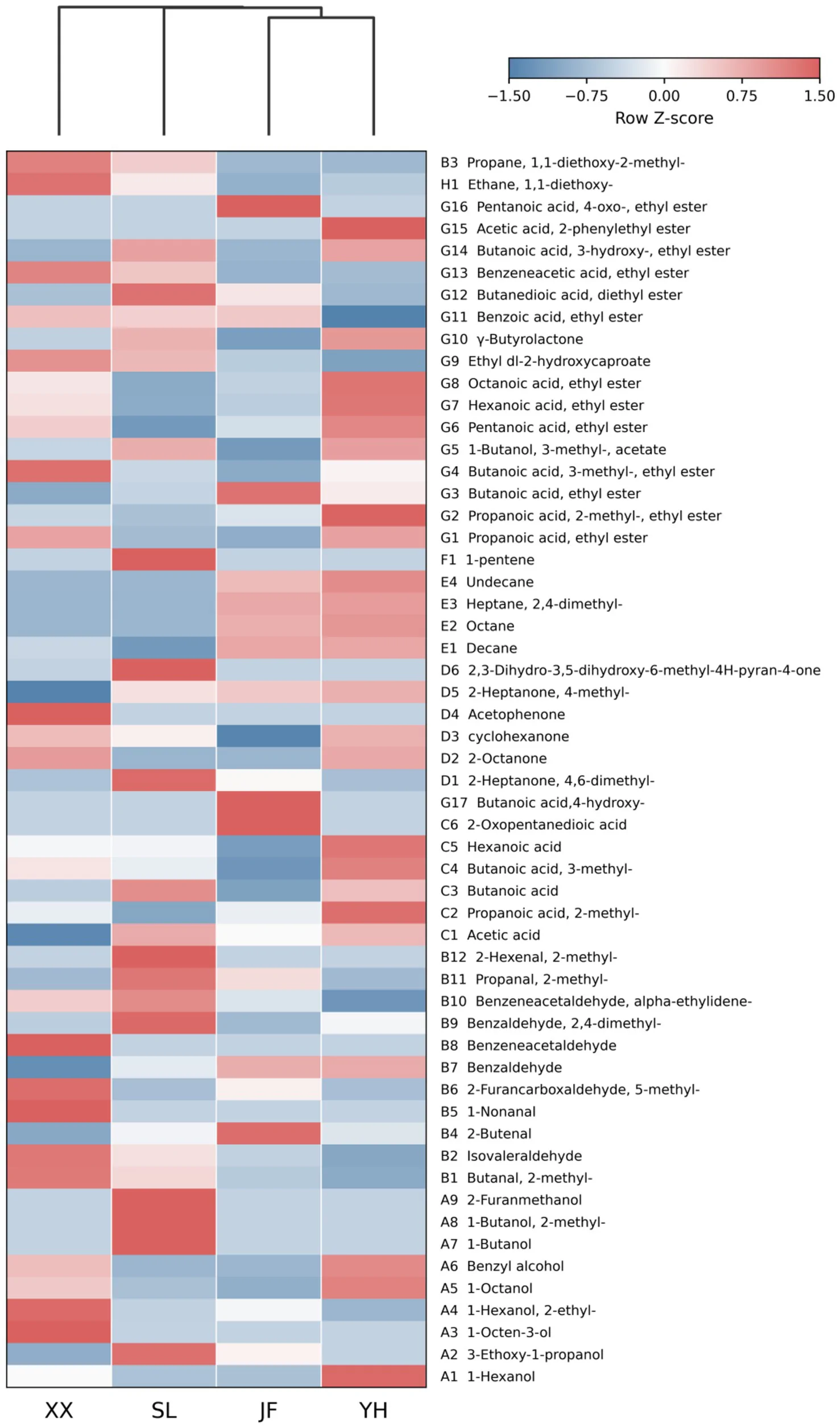
Heatmap of the 56 volatile compounds reported in Table 2 for the four commercial Huangjiu products. Colors represent row-wise *Z*-scores of the mean semi-quantitative concentrations. Entries reported as a dash were assigned zero for heatmap computation; this does not establish a true concentration of zero. Columns were hierarchically clustered using Euclidean distance and average linkage. Rows follow the reverse order of Table 2 and were not clustered. Compound codes correspond to Table 2.

**Table 2:** Volatile organic compounds content (μg/kg; −indicates undetectable).

Type	ID	Retention time(min)	CAS	Compound name	Structural formula	VOC scontent (μg/kg)											
						XX	STD	RSD (%)	SL	STD	RSD (%)	YH	STD	RSD (%)	JF	STD	RSD (%)
Alcohols	A1	24.89	111–27-3	1-Hexanol	C6H14O	112.26	1.87	1.66	72.69	5.06	6.96	193.57	4.18	2.16	71.37	1.60	2.24
	A2	26.045	111–35-3	3-Ethoxy-1-propanol	C5H12O2	7.88	0.98	12.49	23.18	3.26	14.08	10.91	0.58	5.33	14.78	1.53	10.39
	A3	29.474	3,391-86-4	1-Octen-3-ol	C8H16O	3.99	0.63	15.76	–	–	–	–	–	–	–	–	–
	A4	31.539	104–76-7	1-Hexanol, 2-ethyl-	C8H18O	29.28	1.31	4.47	15.58	1.38	8.83	13.16	0.88	6.70	18.86	0.55	2.94
	A5	34.192	111–87-5	1-Octanol	C8H18O	46.18	0.64	1.38	25.76	1.15	4.45	57.85	1.72	2.98	22.42	1.08	4.81
	A6	41.666	100–51-6	Benzyl alcohol	C7H8O	7.02	0.78	11.16	–	–	–	9.37	1.25	13.38	–	–	–
	A7	16.048	71–36-3	1-Butanol	C4H10O	–	–	–	15.03	2.01	13.37	–	–	–	–	–	–
	A8	18.563	137–32-6	1-Butanol, 2-methyl-	C5H12O	–	–	–	1115.11	45.17	4.05	–	–	–	–	–	–
	A9	37.115	98–0-0	2-Furanmethanol	C5H6O2	–	–	–	46.79	6.08	13.00	–	–	–	–	–	–
Aldehydes	B1	7.606	96–17-3	Butanal, 2-methyl-	C5H10O	76.47	2.99	3.92	53.21	5.15	9.68	19.14	2.26	11.79	29.09	0.93	3.20
	B2	7.704	590–86-3	Isovaleraldehyde	C5H10O	155.94	2.46	1.58	109.28	6.29	5.75	48.93	2.64	5.39	73.25	2.40	3.27
	B4	11.305	4,170-30-3	2-Butenal	C4H6O	6.27	0.67	10.61	8.18	0.42	5.08	7.80	0.34	4.42	11.16	0.17	1.54
	B5	26.984	124–19-6	1-Nonanal	C9H18O	15.06	2.22	14.73	–	–	–	–	–	–	–	–	–
	B6	30.608	620–2-0	2-Furancarboxaldehyde, 5-methyl-	C6H6O2	29.69	2.51	8.45	–	–	–	–	–	–	11.89	0.64	5.36
	B7	33.109	100–52-7	Benzaldehyde	C7H6O	863.90	21.84	2.53	939.42	30.23	3.22	1004.54	76.98	7.66	1003.06	38.58	3.85
	B8	36.949	122–78-1	Benzeneacetaldehyde	C8H8O	25.71	1.16	4.50	–	–	–	–	–	–	–	–	–
	B9	40.874	15,764–16-6	Benzaldehyde, 2,4-dimethyl-	C9H10O	85.62	7.63	8.91	176.81	14.46	8.18	108.08	17.17	15.89	73.84	2.22	3.01
	B10	42.776	4,411-89-6	Benzeneacetaldehyde, alpha-ethylidene-	C10H10O	24.47	2.81	11.50	33.64	4.46	13.25	–	–	–	13.77	1.49	10.81
	B11	5.906	78–84-2	Propanal, 2-methyl-	C4H8O	–	–	–	9.16	0.54	5.87	–	–	–	4.81	0.46	9.48
	B12	16.75	28,467–88-1	2-Hexenal, 2-methyl-	C7H12O	–	–	–	20.48	3.59	17.52	–	–	–	–	–	–
Acids	C1	29.254	64–19-7	Acetic acid	C2H4O2	445.51	13.00	2.92	711.84	107.90	15.16	695.35	58.82	8.46	616.56	22.98	3.73
	C2	34.421	79–31-2	Propanoic acid, 2-methyl-	C4H8O2	5.92	0.17	2.90	–	–	–	16.40	1.92	11.69	6.04	0.17	2.87
	C3	36.23	107–92-6	Butanoic acid	C4H8O2	46.62	2.91	6.24	57.69	6.69	11.60	54.52	2.10	3.85	42.97	1.62	3.77
	C4	37.354	503–74-2	Butanoic acid, 3-methyl-	C5H10O2	59.74	3.51	5.87	54.21	5.05	9.32	74.01	1.35	1.83	38.57	1.22	3.16
	C5	40.97	142–62-1	Hexanoic acid	C6H12O2	41.83	6.72	16.06	40.74	6.35	15.59	76.64	11.17	14.57	13.13	1.21	9.20
	C6	11.462	328–50-7	2-Oxopentanedioic acid	C5H6O5	–	–	–	–	–	–	–	–	–	46.18	1.86	4.03
	G17	36.558	591–81-1	Butanoic acid,4-hydroxy-	C4H8O3	–	–	–	–	–	–	–	–	–	17.47	2.70	15.47
Ketone	D1	19.913	19,549–80-5	2-Heptanone, 4,6-dimethyl-	C9H18O	8.02	0.75	9.36	11.25	1.06	9.42	7.93	1.21	15.28	9.13	0.32	3.53
	D2	21.866	111–13-7	2-Octanone	C8H16O	55.50	0.46	0.84	–	–	–	51.49	2.52	4.90	–	–	–
	D3	22.221	108–94-1	Cyclohexanone	C6H10O	13.97	1.71	12.22	12.84	1.77	13.79	14.21	1.79	12.59	9.32	1.31	14.03
	D4	37.17	98–86-2	Acetophenone	C8H8O	11.97	0.96	8.00	–	–	–	–	–	–	–	–	–
	D5	17.997	6,137-6-0	2-Heptanone, 4-methyl-	C8H16O	–	–	–	13.55	1.06	7.84	17.24	2.73	15.85	15.29	1.52	9.93
	D6	47.213	28,564–83-2	4H-Pyran-4-one, 2,3-dihydro-3,5-dihydroxy-6-methyl-	C6H8O4	–	–	–	16.93	0.57	3.39	–	–	–	–	–	–
Alkane	E1	9.886	124–18-5	Decane	C10H22	9.15	0.88	9.60	–	–	–	24.91	4.06	16.31	24.96	3.63	14.53
	E2	5.724	111–65-9	Octane	C8H18	–	–	–	–	–	–	6.31	0.91	14.47	5.48	0.75	13.72
	E3	5.845	2,213-23-2	Heptane, 2,4-dimethyl-	C9H20	–	–	–	–	–	–	9.28	1.16	12.48	8.68	0.99	11.46
	E4	12.973	1,120-21-4	Undecane	C11H24	–	–	–	–	–	–	11.18	1.83	16.35	8.55	0.59	6.85
Olefin	F1	18.453	109–67-1	1-pentene	C5H10	–	–	–	1985.76	94.42	4.75	–	–	–	–	–	–
Esters	G1	8.724	105–37-3	Propanoic acid, ethyl ester	C5H10O2	41.19	0.54	1.31	25.19	2.79	11.06	41.33	2.83	6.84	23.35	1.98	8.47
	G2	8.958	97–62-1	Propanoic acid, 2-methyl-, ethyl ester	C6H12O2	17.97	1.15	6.38	15.08	1.79	11.89	40.10	1.01	2.52	19.99	0.99	4.97
	G3	11.122	105–54-4	Butanoic acid, ethyl ester	C6H12O2	130.85	2.40	1.83	136.54	17.69	12.95	143.52	10.37	7.23	156.51	7.02	4.48
	G4	12.237	108–64-5	Butanoic acid, 3-methyl-, ethyl ester	C7H14O2	29.50	0.31	1.04	18.77	2.49	13.26	21.86	0.47	2.16	15.48	1.23	7.97
	G5	14.303	123–92-2	1-Butanol, 3-methyl-, acetate	C7H14O2	18.74	3.10	16.54	23.27	1.65	7.08	23.76	2.10	8.83	16.11	2.13	13.22
	G6	14.796	539–82-2	Pentanoic acid, ethyl ester	C7H14O2	11.56	1.42	12.30	–	–	–	16.35	1.65	10.07	5.94	0.57	9.53
	G7	19.326	123–66-0	Hexanoic acid, ethyl ester	C8H16O2	92.35	2.38	2.57	49.64	5.68	11.45	127.37	11.90	9.34	63.89	3.03	4.74
	G8	28.909	106–32-1	Octanoic acid, ethyl ester	C10H20O2	17.33	2.09	12.08	15.08	2.22	14.71	19.41	2.77	14.27	15.98	1.67	10.45
	G9	33.793	6,946-90-3	Ethyl dl-2-hydroxycaproate	C8H16O3	182.96	1.06	0.58	165.08	12.98	7.86	80.98	6.37	7.87	106.15	7.37	6.94
	G10	36.592	96–48-0	γ-Butyrolactone	C4H6O2	8.56	1.00	11.70	25.75	2.54	9.87	29.05	4.19	14.44	–	–	–
	G11	37.569	93–89-0	Benzoic acid, ethyl ester	C9H10O2	19.90	1.30	6.51	18.25	2.27	12.42	–	–	–	19.14	2.47	12.92
	G12	37.7	123–25-1	Butanedioic acid, diethyl ester	C8H14O4	488.66	3.81	0.78	956.98	142.21	14.86	464.18	49.90	10.75	699.46	49.42	7.07
	G13	40.173	101–97-3	Benzeneacetic acid, ethyl ester	C10H12O2	24.35	1.10	4.52	20.84	1.83	8.79	13.62	2.06	15.10	12.89	1.85	14.35
	G14	32.838	5,405-41-4	Butanoic acid, 3-hydroxy-, ethyl ester	C6H12O3	–	–	–	15.55	0.58	3.75	15.46	0.94	6.08	–	–	–
	G15	40.744	103–45-7	Acetic acid, 2-phenylethyl ester	C10H12O2	–	–	–	–	–	–	9.44	1.18	12.47	–	–	–
	G16	35.945	539–88-8	Pentanoic acid, 4-oxo-, ethyl ester	C7H12O3	–	–	–	–	–	–	–	–	–	9.82	1.18	12.04
Others	H1	7.132	105–57-7	Ethane, 1,1-diethoxy-	C6H14O2	116.98	5.15	4.40	103.80	10.00	9.63	95.10	9.15	9.62	91.51	3.16	3.45
	B3	9.289	1741-41-9	Propane, 1,1-diethoxy-2-methyl-	C8H18O2	16.10	0.41	2.54	10.22	1.24	12.10	–	–	–	–	–	–

Compound IDs were retained after correction of the chemical classifications to maintain correspondence with the figures.

Variables with VIP values >1 were interpreted as exploratory contributors to separation within the fitted models, rather than confirmed aroma-active compounds or validated markers of sweetness categories. Given the limited sample size, the fitted separation and VIP rankings were interpreted as exploratory findings. Permutation-test results alone were not considered sufficient evidence of predictive performance on independent samples.

## 3 Results and data analysis

### 3.1 Physical and chemical properties of Huangjiu

The total sugar content of YH was 12.5 ± 1.5 g/L with an alcohol content of 15.2 ± 0.2% vol, whereas JF contained 32.6 ± 2.1 g/L of total sugar and 15.1 ± 0.1% vol alcohol. SL contained 68.4 ± 3.5 g/L total sugar and 15.0 ± 0.1% vol alcohol. XX had a total sugar content of 105.3 ± 4.2 g/L and an alcohol content of 17.0 ± 0.2% vol.

The measured total sugar contents were consistent with the respective sweetness classifications specified in GB/T 13662–2018: dry (≤15.0 g/L), semi-dry (15.1–40.0 g/L), semi-sweet (40.1–100.0 g/L), and sweet (>100.0 g/L). Total sugar content, comprising both reducing sugars and non-reducing sugars (e.g., glucose, fructose, maltose, sucrose), is a key parameter for Huangjiu classification and determines its sweetness and overall taste profile. Dry-type Huangjiu contains ≤15.0 g/L total sugar, the lowest among the four categories. Its brewing process emphasizes complete sugar fermentation, yielding a dry, refreshing profile with minimal sweetness. Semi-dry Huangjiu contains 15.1–40.0 g/L total sugar. During fermentation, most sugars are converted to alcohol, with residual sugar imparting a characteristic mellow and mildly sweet taste. This type is the most commonly consumed Huangjiu in China. Alcohol content, a key physicochemical indicator, is produced via anaerobic fermentation of starch and sugars during brewing, directly affecting flavor, mouthfeel, and storage stability.

### 3.2 Analysis of electronic nose results

Figure 2 presents the electronic nose response values for Huangjiu of different sweetness types. The odor radar charts of Huangjiu with varying sweetness profiles were largely similar, possibly due to comparable volatile components or limited sensitivity of the electronic nose sensors to certain compounds. Nevertheless, the electronic nose detected distinct flavor differences among the samples, with sensors S4, S5, S12, S17, and S18 showing pronounced variations. Radar chart analysis indicated that XX exhibited higher responses at sensors S17 and S18, indicating elevated levels of ammonia, amines, toluene, acetone, and ethanol. These distinctive markers differentiate XX from other varieties, likely reflecting the higher sugar content of sweet-type Huangjiu or fermentation byproducts. The stronger responses observed in sensors S17 and S18 suggest differences in the overall volatile composition of XX relative to the other samples. However, because electronic nose sensors exhibit broad and overlapping sensitivities, these responses should be interpreted as indicative aroma patterns rather than direct evidence for specific volatile compounds.

**Figure 2:**
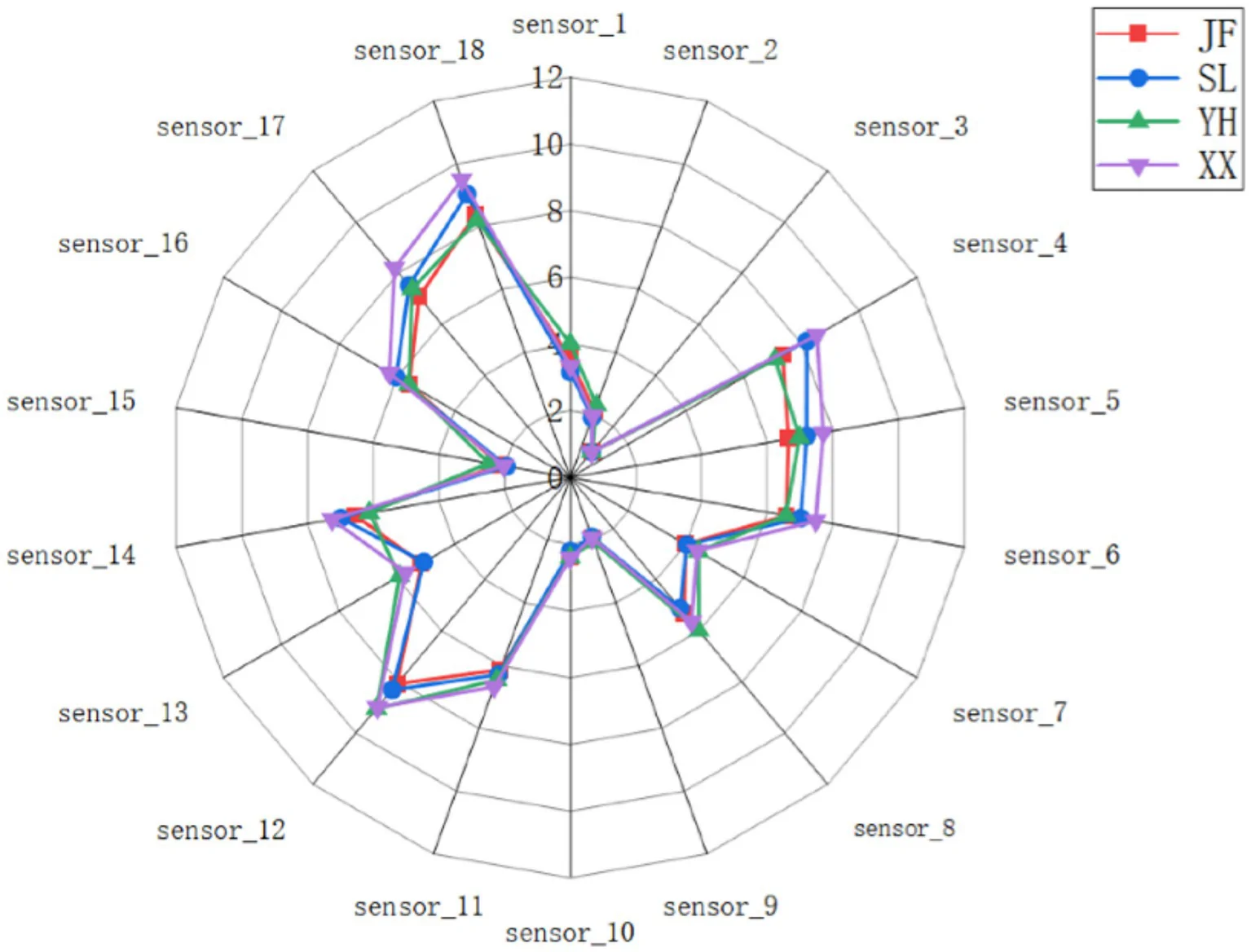
Detection results of electronic nose.

For the remaining three groups, differentiation was achieved through subtle differences across specific sensors. SL showed the highest response at S4 (sulfide), indicating relatively high sulfide content in semi-sweet Huangjiu. The higher response of sensor S4 may reflect differences in sulfur-containing volatile compounds, although the specific chemical contributors cannot be resolved solely from electronic nose measurements. This response pattern suggests that YH possesses a volatile profile different from those of the remaining samples, which was further investigated using GC–MS and GC-IMS. Overall, JF showed lower responses than SL and XX, aligning more closely with YH and reflecting its characteristic flavor profile. Based on sensor responses, SL and XX can be grouped together, whereas YH aligns more closely with JF. Radar chart analysis indicated that SL and XX exhibit the highest odor intensity and most pronounced flavor profiles among the samples. High aldehyde content indicates stronger fruity aromas and aging characteristics, with sulfur-containing compounds (S4, S16) and amines (S17) serving as primary response markers. The primary characteristics of YH likely reflect its lower residual sugar content and higher alcohol levels, typical of dry-type Huangjiu, with sugars consumed or converted during fermentation. Eelectronic nose radar plots revealed compositional differences in volatile compounds among Huangjiu types, directly influencing flavor and sweetness profiles. Overall, the electronic nose rapidly differentiated the four commercial Huangjiu samples at the pattern-recognition level, suggesting differences in their global volatile fingerprints. However, the chemical identities responsible for these differences require confirmation using chromatographic techniques.

OPLS-DA was applied to explore differences in E-nose response patterns among the sampled products, and a permutation test with 200 permutations was performed to assess the fitted separation relative to randomized class labels. The results are presented in Figure 3. Compared with traditional principal component analysis, OPLS-DA enhances separation among groups by decomposing independent variables into components correlated and uncorrelated with dependent variables. Figure 3B shows a *Q*^2^ regression intercept of −0.669 in the 200-permutation test. This intercept should not be interpreted as the original model’s cross-validated *Q*^2^ value and, by itself, does not establish predictive performance.

**Figure 3:**
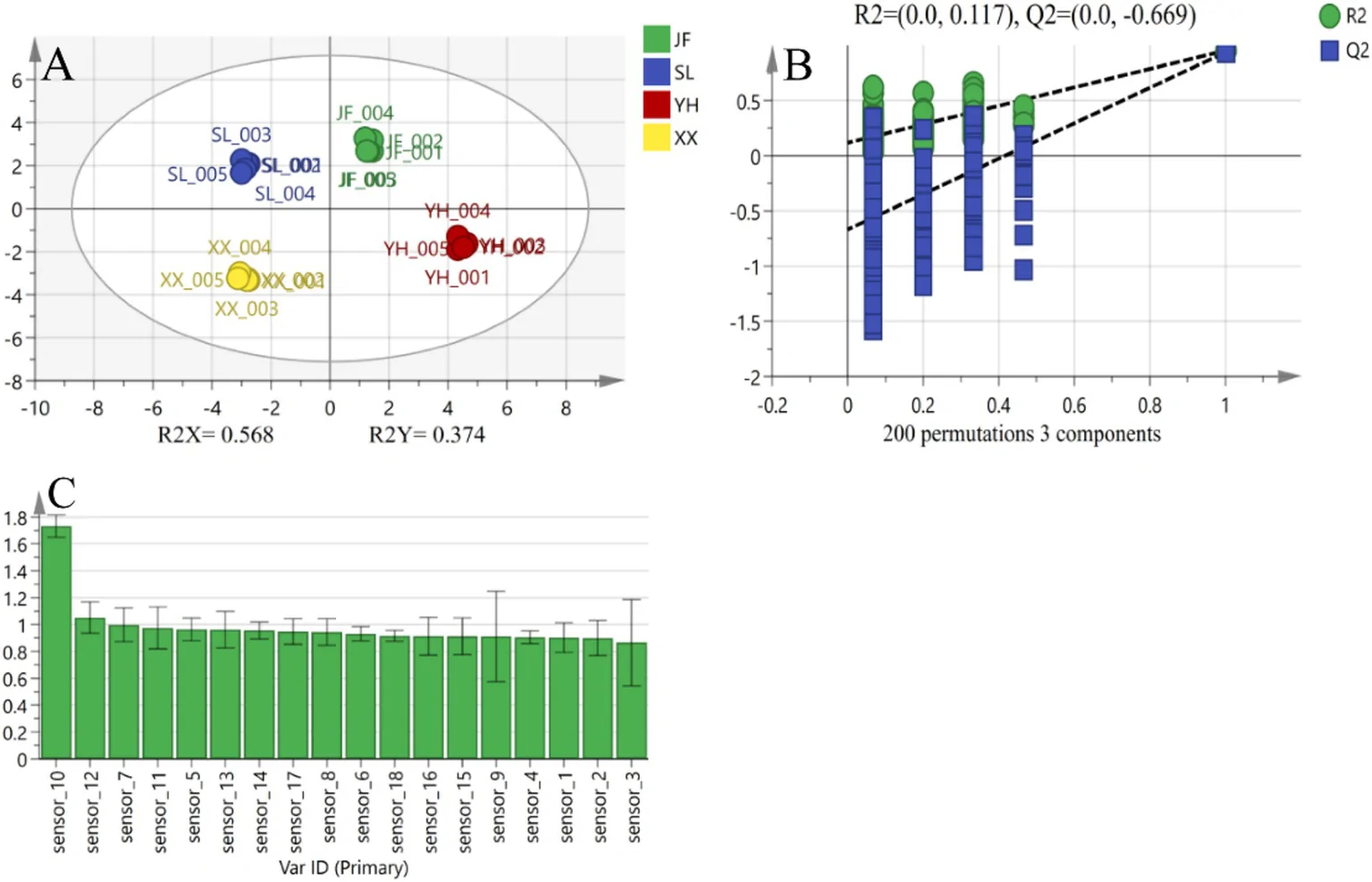
OPLS-DA score plots of electronic nose data and permutation test results (200 permutations). **(A)** Score plots of all samples based on electronic nose data; **(B)** Permutation test of the OPLS-DA model for all samples; **(C)** VIP score derived from electronic nose data for all samples.

The variable importance in VIP plot (Figure 3C) identified sensors with high contributions (e.g., S10), all exhibiting VIP values>1; sensors with VIP values greater than 1 were considered variables contributing to sample discrimination. Sensor S10 had the highest VIP score in the fitted model, followed by S12, S7, and S11. The VIP value of S10 reached 1.73, ranking highest among all sensors. Among the sensors, S10 exhibited the highest VIP value, indicating its relatively greater contribution to sample discrimination within the electronic nose model. Nevertheless, because electronic nose sensors respond to groups of volatile compounds rather than individual analytes, the chemical interpretation of sensor importance should be made with caution. For example, sensor_12, a high-ranking VIP, responds to terpenoids (enriched in SL), whereas sensor_1 is sensitive to short-chain alcohols (higher in YH). These findings establish the chemical basis for inter-group differences. In conclusion, the E-nose provided rapid but preliminary discrimination of aroma patterns. The E-nose results are interpreted as exploratory descriptions of the measured response patterns. The VIP rankings describe contributions within the fitted model and should not be regarded as validated indicators of discrimination among sweetness categories.

Nevertheless, it served as an effective pre-screening tool to guide subsequent targeted analysis by GC–MS and GC-IMS. Because the electronic nose provides global odor fingerprints rather than compound-specific information, its results were interpreted together with GC–MS and GC-IMS analyses in the following sections.

### 3.3 HS-SPME-GC-MS analysis

HS-SPME-GC-MS was employed to characterize the volatile composition of commercial Huangjiu representing four sweetness classifications. Volatile compounds were tentatively identified based on NIST library matching (match factor >90%) and semi-quantified using an internal standard. The analytical results provide detailed compositional information complementary to the global odor patterns obtained by the electronic nose. The concentrations of VOCs were calculated based on the peak areas of detected compounds relative to those of the internal standards and their corresponding concentrations. A total of 56 volatile compounds were tentatively identified across all samples. Although many compounds were shared among the four Huangjiu categories, noticeable differences were observed in both compound occurrence and relative abundance, indicating compositional variation among commercial sweetness classifications. Based on the entries in Table 2, 40 compounds were tentatively identified in XX, comprising 13 esters, 9 aldehydes, 6 alcohols, 5 acids, 4 ketones, 1 alkane, and 2 acetals classified as others. SL contained 39 compounds, comprising 13 esters, 8 aldehydes, 7 alcohols, 4 acids, 4 ketones, 1 alkene, and 2 acetals. YH contained 38 compounds, comprising 14 esters, 5 aldehydes, 5 alcohols, 5 acids, 4 ketones, 4 alkanes, and 1 acetal. JF contained 40 compounds, comprising 13 esters, 8 aldehydes, 4 alcohols, 7 acids, 3 ketones, 4 alkanes, and 1 acetal. Differences in VOC composition and semi-quantitative concentrations were observed among the four commercial Huangjiu products. In terms of total VOC concentration, YH exhibited the highest level (7901.539 μg/kg), followed by SL and JF, whereas XX, showed the lowest concentration (5843.025 μg/kg). These differences reflect the overall compositional characteristics of the analyzed commercial products rather than demonstrating a causal effect of sweetness itself.

Figure 4 shows the summed semi-quantitative concentrations of the volatile compounds in each chemical class, while Table 2 presents the values for individual compounds. Esters represented the dominant class across all commercial Huangjiu samples, followed by aldehydes, organic acids, and alcohols. Although the overall composition was similar among the four sweetness classifications, noticeable differences in the relative abundance of individual compound classes were observed, indicating distinct volatile fingerprints among the samples.

**Figure 4:**
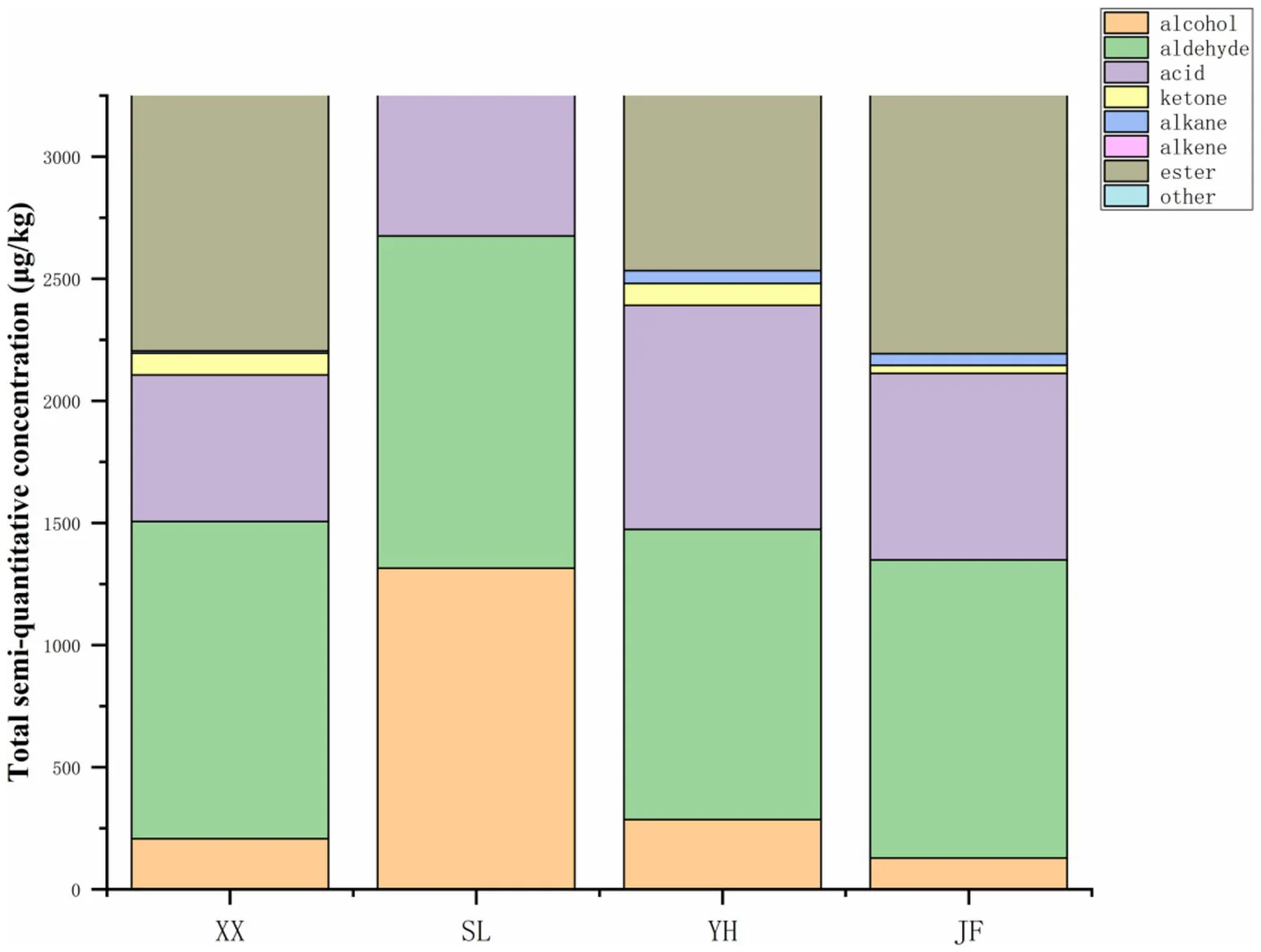
Summed semi-quantitative concentrations of volatile compounds by chemical class in the four commercial Huangjiu samples, determined by HS-SPME-GC–MS. Each colored segment represents the sum of the semi-quantitative concentrations of compounds assigned to that class.

B7 (benzaldehyde) was tentatively identified in all four commercial Huangjiu products. Benzaldehyde formation may arise from the degradation of phenylalanine by yeast via the Ehrlich pathway during fermentation. Alternatively, it may be generated through Maillard reactions with reducing sugars during brewing, followed by Strecker degradation. At appropriate concentrations, benzaldehyde imparts fruity, sweet, nutty, and caramel-like aromas (27, 28). These interactions may contribute to the modulation and balance of other flavor compounds in Huangjiu, thereby shaping its characteristic sensory profile.

F1 (1-pentene) was tentatively identified only in the SL sample under the present analytical conditions. Confirmation using an authentic standard and analysis of additional independent production batches are required before this observation can be considered characteristic of semi-sweet Huangjiu.

Based on the quantitative data of volatile components, OPLS-DA was performed to evaluate differences in VOC profiles among different Huangjiu samples (Figure 5A). The analyzed samples showed separation in the fitted model, which was interpreted as exploratory rather than evidence of predictive performance for independent samples. The VIP value represents the contribution and explanatory power of each volatile component to sample classification. VIP values describe the relative contributions of variables to the fitted model; a VIP value >1 does not, by itself, establish a statistically significant between-group difference. The VIP scores of individual variables are presented in Figure 5C. These variables were interpreted as exploratory indicators of differences in volatile composition rather than confirmed key aroma compounds, because sensory evaluation, odor activity value calculations, and GC–olfactometry were not performed in this study.

**Figure 5:**
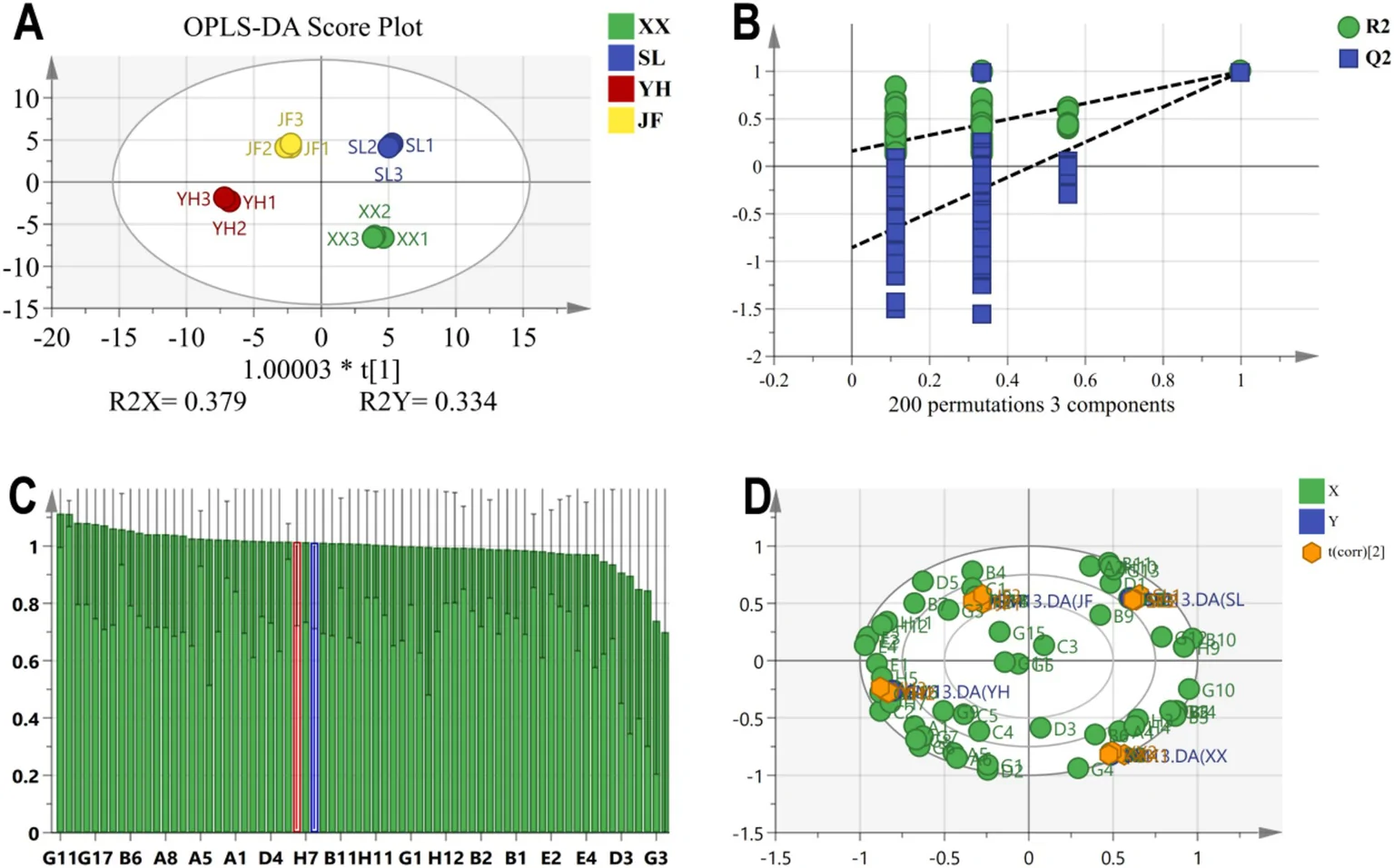
OPLS-DA score plots and permutation test results based on HS-SPME-GC–MS data. **(A)** Score plots based on HS-SPME-GC–MS data; **(B)** OPLS-DA permutation test for HS-SPME-GC–MS data; **(C)** Calculated VIP scores for volatile compounds; **(D)** Dual labeling plots of volatile compounds and samples.

As shown in Figure 5A, the four Huangjiu samples were distributed across distinct quadrants. The SL sample was mainly associated with several alcohols, aldehydes, and Maillard-related compounds exhibiting VIP values >1, suggesting that these compounds contributed substantially to differentiation of the SL volatile profile. The SL sample was located in the first quadrant, in close proximity to A7 (n-butanol), A8 (2-methylbutanol), A9 (2-furanmethanol), B12 (2-methylhexenal), D6 (2,3-dihydro-3,5-dihydroxy-6-methyl-4H-pyrano-4-one), and F1 (1-pentene). All of these compounds exhibited VIP values >1. This indicates that these compounds are differential volatile contributors to the volatile profile of the SL sample. The JF sample was located in the second quadrant, in proximity to C1 (acetic acid) which exhibited VIP values <1. This indicates that although acetic acid is present at a relatively high concentration in JF, its contribution to sample discrimination is limited. The YH and XX samples were located in the third and fourth quadrants, respectively. Differential volatile compounds associated with the YH sample (VIP > 1 and spatial proximity) included G2 (ethyl 2-methylpropionate) and G12 (diethyl succinate). The primary volatile components contributing to the XX sample included A3 (1-octen-3-ol), B5 (nonanal) and B8 (benzeneacetaldehyde) all of which exhibited VIP values>1.

A hierarchical clustering heatmap was used to visualize variation in the volatile profiles of the four commercial Huangjiu samples (Figure 1). The heatmap summarizes differences in the measured volatile profiles across the samples and complements the compound-level results presented in Table 2. Because the analysis relied on tentative identification and semi-quantitative measurements, and no sensory validation was performed, these patterns should be interpreted as compositional differences rather than evidence of specific sensory contributions.

### 3.4 GC-IMS data analysis of different sweet types of Huangjiu

The peaks in total ion chromatograms were extracted using a chromatography data system and matched against standard databases, including LRI/odour/Flavornet, Human odor space, the Flavor Ingredient Library, and the Good Scents Company Information System. Table 3 lists 77 signal peaks, comprising 72 peaks tentatively assigned to 54 sample volatile compounds, two peaks corresponding to the monomer and dimer of the added 2-octanol internal standard, and three unidentified peaks. Monomer and dimer signals assigned to the same compound were counted as one compound. The internal standard and unidentified peaks were excluded from the count of 54 compounds. As shown in Table 3, certain compounds were detected as proton-bound dimers and monomers, likely resulting from interactions among volatile compounds. Such interactions may produce multiple signal forms, including dimers and trimers.

**Table 3:** Qualitative results of GC-IMS data.

Type	ID	Compound	CAS#	Formula	MW	RI	Rt (s)	Dt (RIPrel)
Alcohol	A1	3-Methyl-1-butanol-M	C123513	C5H12O	88.1	1228.8	702.86	1.24797
	A2	3-Methyl-1-butanol-D	C123513	C5H12O	88.1	1228.8	702.86	1.4932
	A3	1-Butanol-M	C71363	C4H10O	74.1	1168.3	601.162	1.26683
	A4	1-Butanol-D	C71363	C4H10O	74.1	1168.6	601.831	1.38778
	A5	2-Methyl-1-propanol	C78831	C4H10O	74.1	1116.9	504.147	1.37447
	A6	1-Propanol	C71238	C3H8O	60.1	1,065	429.388	1.24971
	A7	Ethanol	C64175	C2H6O	46.1	954.5	321.403	1.13732
	A8	Methanol	C67561	CH4O	32	903.7	287.697	0.97794
	A9	cis-2-Penten-1-ol	C1576950	C5H10O	86.1	1311.1	832.819	0.95753
	A10	1-Hexanol	C111273	C6H14O	102.2	1379.4	965.35	1.40352
	A11	2-Butanol	C78922	C4H10O	74.1	1032.7	391.253	1.32021
	A12	2-Octanol-M(internal standard)	C123966	C8H18O	130.2	1455.4	1137.435	1.42814
	A13	2-Octanol-D(internal standard)	C123966	C8H18O	130.2	1455.7	1138.335	1.83658
Aldehyde	B1	Citronellal	C106230	C10H18O	154.3	1504.6	1265.249	1.21162
	B2	3-Methylbutanal	C590863	C5H10O	86.1	944.9	314.769	1.41568
	B3	2-Methyl-2-propenal	C78853	C4H6O	70.1	904.2	287.966	1.20594
	B4	2-Methylpropanal	C78842	C4H8O	72.1	847.6	254.517	1.28135
	B5	Propanal	C123386	C3H6O	58.1	834.3	247.234	1.06932
	B6	Pentanal	C110623	C5H10O	86.1	1004.4	360.529	1.40999
	B7	Benzaldehyde-M	C100527	C7H6O	106.1	1560.5	1427.659	1.28007
	B8	Benzaldehyde-D	C100527	C7H6O	106.1	1,561	1429.192	1.47499
	B9	Hexenal	C1335393	C6H10O	98.1	1109.3	491.146	1.30003
Acid	C1	Acetic acid	C64197	C2H4O2	60.1	1508.2	1275.015	1.15631
	C2	3-Methylbutanoic acid	C503742	C5H10O2	102.1	1720	2014.849	1.20234
Ketone	D1	3-Hydroxy-2-butanone-M	C513860	C4H8O2	88.1	1,305	821.954	1.20913
	D2	3-Hydroxy-2-butanone-D	C513860	C4H8O2	88.1	1,305	821.954	1.33119
	D3	2-Pentanone	C107879	C5H10O	86.1	1000.4	356.415	1.09215
	D4	Acetone	C67641	C3H6O	58.1	852.9	257.484	1.1119
	D5	2-Butanone	C78933	C4H8O	72.1	930.4	304.961	1.24409
	D6	2-Heptanone-M	C110430	C7H14O	114.2	1200.3	663.226	1.3446
	D7	2-Heptanone-D	C110430	C7H14O	114.2	1200.3	663.226	1.63377
	D8	2,3-Pentanedione	C600146	C5H8O2	100.1	1074.8	441.678	1.20619
	D9	3-Penten-2-one	C625332	C5H8O	84.1	1125.1	518.593	1.3535
Ester	E1	Ethyl octanoate	C106321	C10H20O2	172.3	1460.5	1150.232	1.48819
	E2	Ethyl lactate-M	C97643	C5H10O3	118.1	1367.5	940.814	1.30565
	E3	Ethyl lactate-D	C97643	C5H10O3	118.1	1367.5	940.814	1.53798
	E4	Ethyl hexanoate-M	C123660	C8H16O2	144.2	1253.4	738.989	1.34673
	E5	Ethyl hexanoate-D	C123660	C8H16O2	144.2	1253.8	739.658	1.79836
	E6	Isoamyl acetate-M	C123922	C7H14O2	130.2	1146.3	557.672	1.31788
	E7	Isoamyl acetate-D	C123922	C7H14O2	130.2	1146.7	558.341	1.75064
	E8	Ethyl 3-methylbutanoate-M	C108645	C7H14O2	130.2	1094.6	467.717	1.27597
	E9	Ethyl 3-methylbutanoate-D	C108645	C7H14O2	130.2	1095.2	468.454	1.65623
	E10	Ethyl 2-methylbutanoate-M	C7452791	C7H14O2	130.2	1078.1	445.973	1.25812
	E11	Ethyl 2-methylbutanoate-D	C7452791	C7H14O2	130.2	1078.1	445.973	1.65098
	E12	Ethyl butanoate	C105544	C6H12O2	116.2	1063.5	427.545	1.56064
	E13	2-Methylpropyl acetate-M	C110190	C6H12O2	116.2	1039.7	399.167	1.34215
	E14	2-Methylpropyl acetate-D	C110190	C6H12O2	116.2	1039.7	399.167	1.61316
	E15	Propyl acetate-M	C109604	C5H10O2	102.1	1005.4	361.575	1.28333
	E16	Propyl acetate-D	C109604	C5H10O2	102.1	1005.4	361.575	1.48186
	E17	Ethyl isobutyrate-M	C97621	C6H12O2	116.2	994.7	350.887	1.32429
	E18	Ethyl isobutyrate-D	C97621	C6H12O2	116.2	995.6	351.624	1.56484
	E19	Ethyl propanoate-M	C105373	C5H10O2	102.1	988.4	346.096	1.27072
	E20	Ethyl propanoate-D	C105373	C5H10O2	102.1	988.4	346.096	1.45245
	E21	Ethyl acetate	C141786	C4H8O2	88.1	912.1	293.025	1.33585
	E22	Ethyl formate	C109944	C3H6O2	74.1	859.6	261.261	1.22635
	E23	Methyl acetate	C79209	C3H6O2	74.1	857.7	260.182	1.174
	E24	(Z)-3-Hexen-1-yl acetate	C3681718	C8H14O2	142.2	1,312	834.552	1.3041
	E25	Hexyl acetate	C142927	C8H16O2	144.2	1280.4	780.806	1.36573
	E26	Isoamyl butyrate	C2050013	C9H18O2	158.2	1202.8	666.586	1.37129
	E27	Ethyl (E)-2-butenoate-M	C623701	C6H10O2	114.1	1186.5	639.921	1.30563
	E28	Ethyl (E)-2-butenoate-D	C623701	C6H10O2	114.1	1186.5	639.921	1.55714
	E29	Butyl isobutyrate-M	C97870	C8H16O2	144.2	1,178	621.6	1.30356
	E30	Butyl isobutyrate-D	C97870	C8H16O2	144.2	1,178	621.6	1.80761
	E31	Ethyl pentanoate-M	C539822	C7H14O2	130.2	1,159	582.318	1.38356
	E32	Ethyl pentanoate-D	C539822	C7H14O2	130.2	1159.2	582.79	1.68271
	E33	2-Methylbutyl acetate	C624419	C7H14O2	130.2	1134.8	536.051	1.2993
	E34	Butyl acetate	C123864	C6H12O2	116.2	1098.1	472.636	1.23949
	E35	Propyl propionate	C106365	C6H12O2	116.2	1053.3	415.189	1.58657
	E36	Methyl butanoate	C623427	C5H10O2	102.1	1010.6	366.997	1.44027
Other	F1	Furfural-M	C98011	C5H4O2	96.1	1503.8	1263.079	1.09269
	F2	Tetrahydrofuran	C109999	C4H8O	72.1	878.1	272.051	1.2574
	F3	Linalool	C78706	C10H18O	154.3	1643.2	1706.689	1.22439
	F4	Furfural-D	C98011	C5H4O2	96.1	1503.5	1262.081	1.3462
	F5	2-Methylthiophene	C554143	C5H6S	98.2	1121.5	512.21	1.02761
Unknown	G1	1	Unidentified	*	0	1097.3	471.359	1.52704
	G2	2	Unidentified	*	0	1076.5	443.912	1.45541
	G3	3	Unidentified	*	0	1,069	434.338	1.63197

M and D denote monomer and dimer signals, respectively. A12 and A13 correspond to the added 2-octanol internal standard and were excluded from both the sample compound count and the chemometric analysis. Monomer and dimer signals of the same compound were counted once when determining the number of compounds but were retained as separate variables for chemometric analysis. G1–G3 denote unidentified peaks.

To facilitate intuitive comparison of volatile profiles among samples, the spectrum of sample XX was selected as the reference, and spectra of other samples were subtracted to obtain differential plots, as shown in Figure 6. When the target and reference samples contain identical levels of volatile organic compounds (VOCs), the resulting background appears white. Red indicates a higher concentration of a compound in the target sample than in the reference, whereas blue indicates a lower concentration. As shown in Figure 6, the volatile profiles of YH and JF samples are similar, as are those of XX and SL samples. This pattern is likely associated with differences in sweetness levels and sugar content.

**Figure 6:**
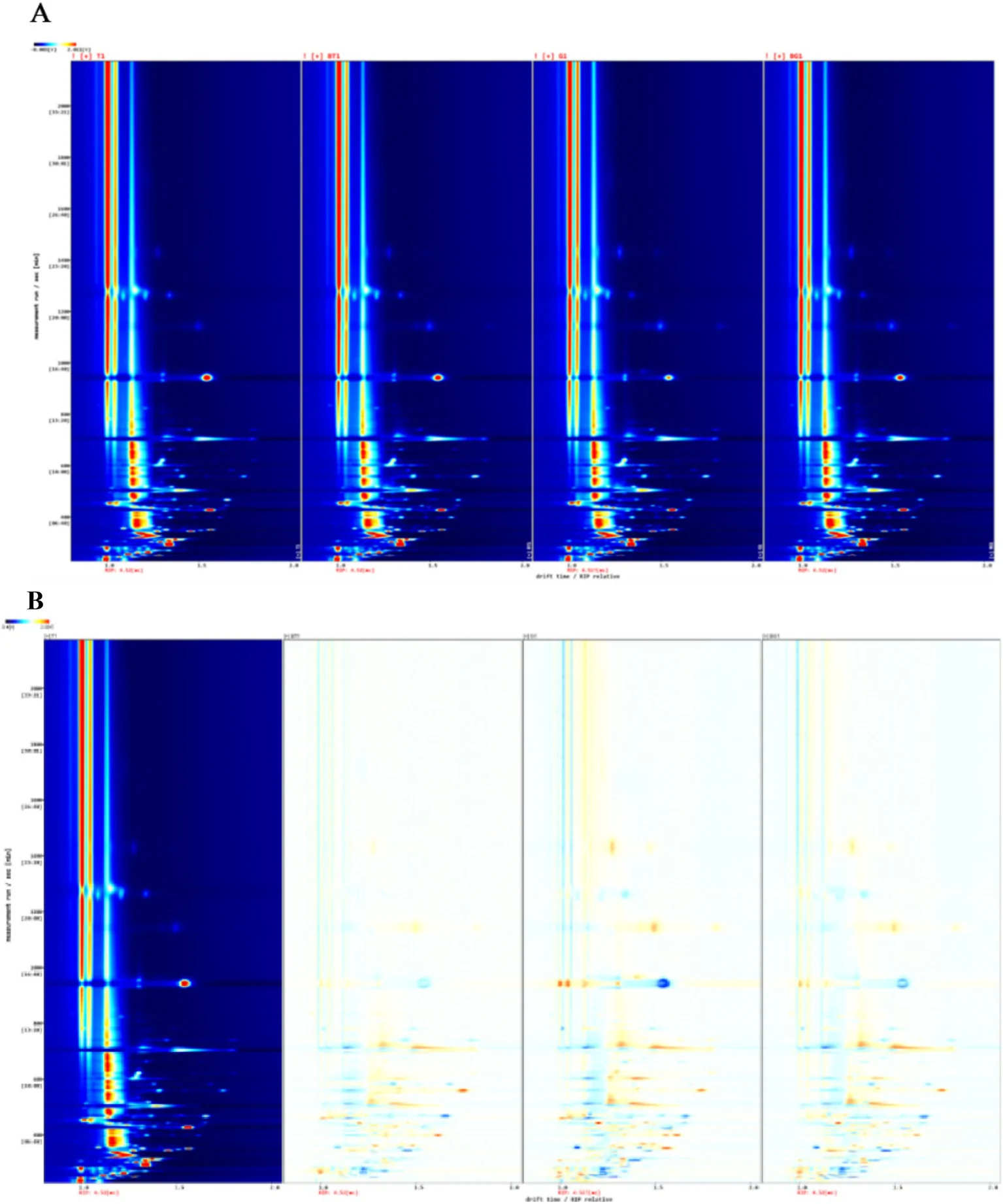
GC-IMS results **(A)** Top view **(B)** Differential spectrum.

The fingerprint spectrum shown in Figure 7 enables visual comparison of the overall VOC profiles of each sample group and the differences among them. Each row in the figure represents either an individual sample or the complete set of signal peaks obtained during analysis. As shown in Figure 7, marked differences in volatile components were observed among the four sweetness types of Huangjiu, while intra-group similarities were also evident. Overall, the GC-IMS fingerprints showed differences in volatile profiles among the four commercial Huangjiu products.

**Figure 7:**
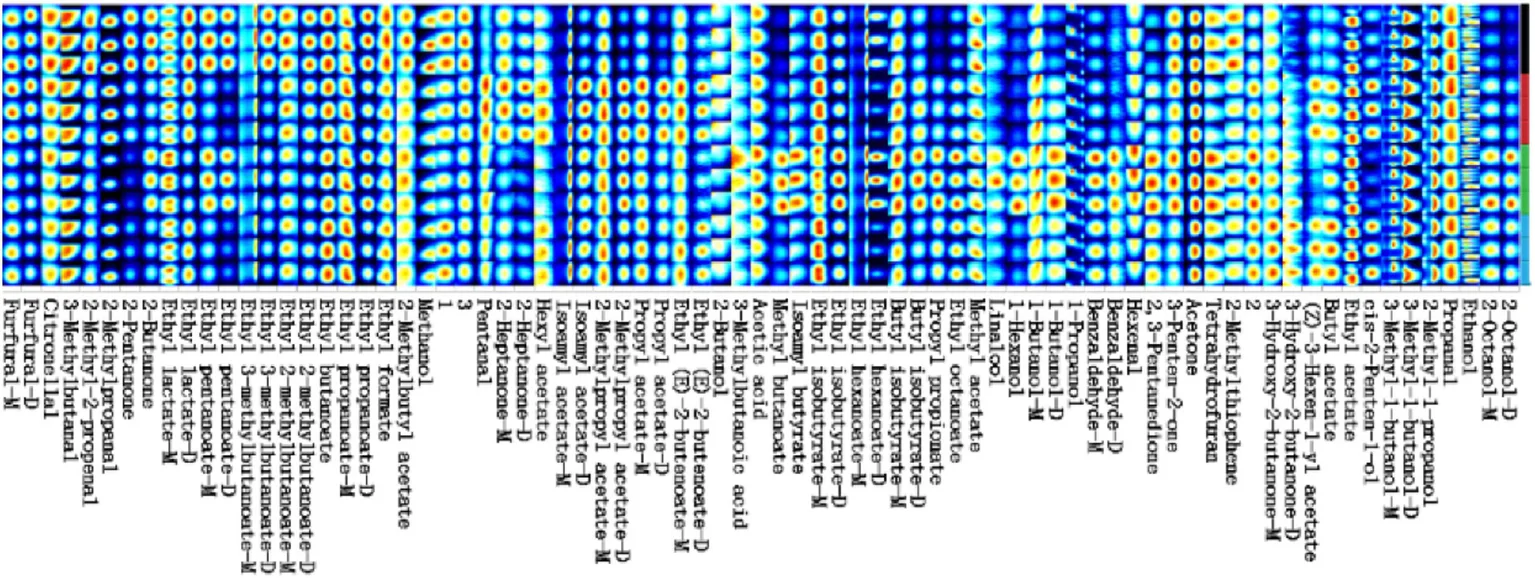
Volatile component fingerprint profiles in Huangjiu samples with different sweetness levels.

Further analysis of volatile components revealed that the XX sample contained higher concentrations of furfural, citronellal, 3-methylbutyraldehyde, 2-methyl-2-propenal, 2-methylpropanal, 2-pentanone, 2-butanone, ethyl lactate, ethyl valerate, ethyl 3-methylbutyrate, ethyl 2-methylbutyrate, ethyl butyrate, ethyl propionate, ethyl formate, 2-methylbutyl acetate, and methanol. Furfural has been associated with almond-like and caramel notes in soy sauce-aroma baijiu (29). These reported descriptors provide context for interpreting the volatile profile of XX; however, the sensory contributions of the compounds detected here were not established in the present study (30). The ratio of ethyl lactate to ethyl acetate is a key indicator for differentiating aroma types of China liquor (such as light-aroma and strong-aroma types) (31–33).

The SL sample exhibited relatively high concentrations of valeraldehyde, 2-heptanone, hexyl acetate, isopentyl acetate, isobutyl acetate, propyl acetate, ethyl E-2-butenoate, and 2-butanol. Research on rice wine fermented with mixed mould and yeast starters indicates that starter composition can influence flavour characteristics (34). However, the present study did not establish the microbial origins or specific sensory contributions of the compounds detected in SL (35). The relatively high GC-IMS signals of ethyl (E)-2-butenoate, isoamyl acetate, hexyl acetate, isobutyl acetate, and propyl acetate characterize the ester profile of the analyzed SL product. However, these compositional observations alone do not establish a ripe-fruit aroma or a sensory balance among sweetness, fruitiness, and mellowness. Whether SL and XX differ in perceived fruity or caramel-like notes requires sensory validation.

The contents of 3-methylbutyric acid, acetic acid, methyl butyrate, isopentyl butyrate, ethyl isobutyrate, ethyl hexanoate, butyl isobutyrate, propyl propionate, ethyl octanoate, methyl acetate, linalool, 1-hexanol, 1-butanol, 1-propanol, benzaldehyde, hexanal, 2,3-pentanediol, 3-penten-2-one, and acetone are relatively high in YH. The relatively high GC-IMS signals of acetic acid and 3-methylbutyric acid contributed to the observed acid profile of YH. These measurements do not establish that these compounds produced a refreshing or crisp taste, nor do they directly indicate the intensity of microbial activity during fermentation. Likewise, the detected esters, aldehydes, and ketones describe the volatile composition of YH, while their combined sensory effects remain unverified. Previous research on Huangjiu has shown that yeast-assimilable nitrogen can influence higher alcohol production during fermentation. This provides a possible fermentation-related context for interpreting alcohol profiles, but neither the underlying mechanism nor the contribution of these alcohols to mouthfeel was established in the present study (36–38).

The contents of 3-hydroxy-2-butanone, glycerol acetate, butyl acetate, ethyl acetate, Z-2-penten-1-ol, 3-methyl-1-butanol, 2-methyl-1-propanol, and acetaldehyde are relatively high in JF. The relatively high GC-IMS signals of the listed alcohols and esters characterize the volatile profile of the analyzed JF product. These observations do not establish enhanced freshness, greater aroma complexity, or reduced pungency, because the sensory effects of these compounds were not evaluated. Acetoin (3-hydroxy-2-butanone) has been reported as a butter-aroma compound. Its relatively high GC-IMS signal in JF provides a basis for further sensory investigation, but does not establish a stronger buttery aroma in this product (39–41).

Aroma interpretation should also consider interactions among compounds. Yu et al. combined GC–olfactometry and sensory analysis to investigate ester interactions in Huangjiu and demonstrated synergistic aroma enhancement between ethyl lactate and ethyl acetate (24). The present study also detected these esters, but focused on comparing volatile profiles among commercial products rather than determining their sensory interactions. Thus, their occurrence in our samples provides compositional context for the earlier findings, but does not establish that the same synergistic effects occurred under the different compositions and matrix conditions of the products examined here. In the present study, potential masking or synergistic effects, as well as the influence of ethanol, acids, and other matrix constituents, were not evaluated. Therefore, the reported fruity or creamy descriptors should be regarded as literature-based context rather than confirmed sensory attributes of the samples.

#### 3.4.1 OPLS-DA based on GC-IMS data

OPLS-DA was performed using 75 GC-IMS peak variables, comprising 72 assigned peaks and three unidentified peaks, with sample group as the response variable (Figure 8). The two internal standard peaks were excluded, while monomer and dimer signals were retained as separate variables. The score plot illustrates the distribution of the analyzed samples in the model space (Figure 8A). A permutation test with 200 permutations was performed (Figure 8C), yielding a Q^2^ regression intercept of −0.793. This intercept is not the original model’s cross-validated Q^2^ value and, by itself, does not establish predictive reliability. The observed sample separation is therefore interpreted as exploratory. The VIP scores of the 75 peak variables are shown in Figure 8D. A total of 32 compounds with VIP values >1 were identified, including 18 esters, 6 ketones, 3 alcohols, 3 aldehydes, 1 ether, and 1 unknown substance. The biplot of compounds and samples is shown in Figure 8B. The dominant volatile component in the XX sample was B3 (2-methyl-2-propenal). The differential volatile compounds in the YH sample include A10 (1-hexanol) and E4 (ethyl hexanoate-M).

**Figure 8:**
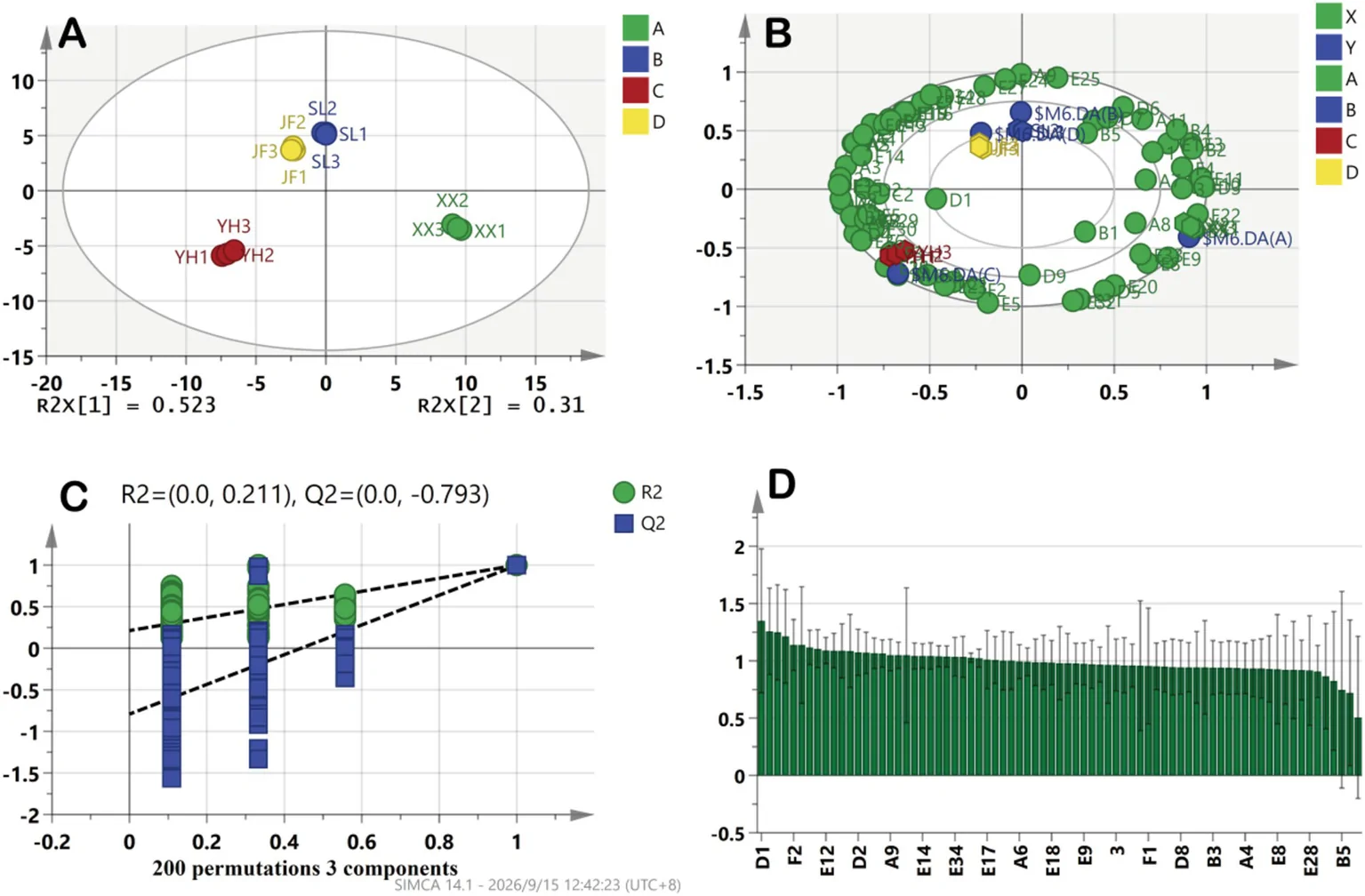
OPLS-DA score plots and permutation test results based on GC-IMS data **(A)** Score plot; **(B)** Biplot of samples and variables; **(C)** Permutation test with 200 permutations; **(D)** Variable importance in projection (VIP) scores.

### 3.5 Comparative analysis of HS-SPME-GC-MS and GC-IMS data

HS-SPME-GC–MS and GC-IMS exhibited complementary analytical capabilities for volatile characterization of commercial Huangjiu samples with different sweetness classifications. A total of 56 volatile compounds were tentatively identified by HS-SPME-GC–MS, whereas GC-IMS tentatively identified 54 volatile compounds, excluding the internal standard. Although considerable overlap was observed between the two analytical platforms, each technique also detected unique compounds because of their distinct analytical principles. These results indicate that neither method alone can comprehensively characterize the volatile composition of Huangjiu, highlighting the value of their combined application.

The larger number of compounds detected by HS-SPME-GC–MS can be primarily attributed to its superior chromatographic separation coupled with mass spectral identification, which provides broader chemical coverage and facilitates the characterization of structurally diverse volatile compounds, particularly medium- and high-boiling constituents. In contrast, GC-IMS relies on atmospheric-pressure chemical ionization and ion mobility separation, making it particularly sensitive to highly volatile, low-molecular-weight compounds. Furthermore, the visual fingerprint generated by GC-IMS enables rapid comparison of volatile profiles among different samples, providing an intuitive approach for sample discrimination. Consequently, HS-SPME-GC–MS and GC-IMS should be regarded as complementary analytical techniques rather than competing methods.

Despite differences in compound coverage, both analytical platforms consistently detected several representative volatile compounds, including acetic acid, benzaldehyde, ethyl butyrate, 3-methylbutyraldehyde, n-hexanol, and n-butanol. The consistent detection of these compounds across two independent analytical platforms increases confidence in their occurrence within the analyzed Huangjiu samples. However, it should be emphasized that these compounds were identified as differential volatile compounds contributing to sample discrimination rather than aroma-active compounds, as no odor activity value (OAV), gas chromatography–olfactometry (GC-O), or sensory validation was performed in the present study.

Comparison of the differential volatile profiles further demonstrated the complementary advantages of the two techniques. HS-SPME-GC–MS provided more comprehensive characterization of relatively less volatile compounds, including aromatic compounds, medium-chain esters, organic acids, and other structurally complex constituents, thereby facilitating detailed compositional analysis. By contrast, GC-IMS showed greater sensitivity toward highly volatile aldehydes, alcohols, ketones, and short-chain esters, while simultaneously providing intuitive two-dimensional topographic plots and fingerprint spectra that clearly visualized differences among Huangjiu samples. Therefore, GC-IMS is particularly suitable for rapid quality evaluation and preliminary discrimination of commercial Huangjiu products, whereas HS-SPME-GC–MS offers more comprehensive compound-level characterization for subsequent chemical interpretation.

Overall, integrating HS-SPME-GC–MS with GC-IMS substantially improves the completeness and reliability of volatile fingerprint characterization compared with either technique alone. The complementary information obtained from the two analytical platforms enables more comprehensive evaluation of compositional differences among commercial Huangjiu samples with different sweetness classifications while reducing the limitations associated with a single analytical method. Nevertheless, the study included one commercial product per sweetness classification from a single manufacturer. The three independent production batches per product provide batch-level replication for the sampled products, but do not establish representativeness across manufacturers or products within each sweetness category. The findings therefore provide a comparative characterization of the four selected commercial products, rather than evidence that the observed differences are caused by sweetness classification alone. In the present comparison, total sugar content varied together with product identity, and XX also had a higher measured alcohol content than the other products. Differences in fermentation conditions, raw materials, and maturation history could also contribute to the observed volatile profiles, but their individual contributions could not be separated from those of sugar content within the present design. For HS-SPME-GC–MS, compound identification relied primarily on mass spectral library matching and should therefore be regarded as tentative rather than confirmed identification using authentic standards. Concentrations estimated relative to the 2-octanol internal standard are semi-quantitative, because this approach does not fully account for compound-specific differences in extraction efficiency and detector response. Matrix differences among products may also affect headspace partitioning and extraction. Accordingly, the reported values support exploratory comparisons of volatile profiles but should not be interpreted as validated absolute concentrations. Further studies incorporating multiple products within each sweetness category from different manufacturers, additional production batches, authentic standards, and sensory validation would help establish the broader applicability and sensory relevance of these findings.

## 4 Conclusion

This study systematically compared the volatile fingerprints of commercial Huangjiu representing the four official sweetness classifications by integrating electronic nose, HS-SPME-GC–MS, and GC-IMS. The three analytical techniques provided complementary information for volatile characterization. Electronic nose enabled rapid discrimination of overall aroma patterns, HS-SPME-GC–MS provided comprehensive compound-level characterization with broader chemical coverage, and GC-IMS facilitated intuitive fingerprint visualization together with high sensitivity toward highly volatile compounds.

Comparative analysis demonstrated reproducible differences in volatile composition among commercial Huangjiu samples with different sweetness classifications. Although numerous volatile compounds were shared among samples, variations in compound occurrence and relative abundance enabled effective discrimination of the four commercial categories. Differential volatile compounds identified in this study provide useful chemical information for comparative characterization, although their aroma contribution requires further validation by odor activity evaluation and sensory analysis.

Overall, integrating electronic nose, HS-SPME-GC–MS, and GC-IMS substantially improved volatile fingerprint characterization compared with the use of a single analytical technique. This integrated analytical strategy provides a practical approach for chemical comparison, quality evaluation, and authenticity assessment of commercial Huangjiu products. Nevertheless, because the present study investigated products from a single manufacturer and compound identification was primarily based on spectral library matching, future studies should include multiple manufacturers, production batches, retention index confirmation, authentic standards, and sensory validation to further strengthen the robustness and applicability of the proposed analytical strategy.

## Data Availability

The original contributions presented in the study are included in the article/supplementary material, further inquiries can be directed to the corresponding authors.
